# Two cases of acrometastasis to the hands and review of the literature

**DOI:** 10.3747/co.v15i5.189

**Published:** 2008-10

**Authors:** C. J. Flynn, C. Danjoux, J. Wong, M. Christakis, J. Rubenstein, A. Yee, D. Yip, E. Chow

**Affiliations:** * Rapid Response Radiotherapy Program, Department of Radiation Oncology, Toronto–Sunnybrook Regional Cancer Centre, Toronto, ON; † Department of Pathology, Toronto–Sunnybrook Regional Cancer Centre, Toronto, ON; ‡ Department of Radiology, Toronto–Sunnybrook Regional Cancer Centre, Toronto, ON; § Department of Orthopedic Surgery, Toronto–Sunnybrook Regional Cancer Centre, Toronto, ON

**Keywords:** Acrometastasis, hand metastasis, bone metastases

## Abstract

This paper reports two cases of acrometastasis to the hands. The first case involved a 78-year-old woman with a permeative osteolytic lesion in her proximal second metacarpal. A biopsy of this lesion suggested a diagnosis of non-small-cell lung carcinoma with secondary osseous metastasis. This was the first presentation of the woman’s primary diagnosis. A single 8-Gy fraction of palliative radiotherapy was delivered to the patient’s left hand. The treatment proved successful: the woman soon experienced pain relief and regained the use of her hand. The second case involved a 69-year-old woman with extensive lytic destruction involving the proximal two thirds of her third metacarpal. This patient had been diagnosed with carcinoma of the breast in 1990. She also received a single 8-Gy fraction of radiation, which improved both her pain and her hand mobility.

An extensive review of the literature uncovered 257 previously reported cases of acrometastasis. Articles were analyzed based on age and sex of the patient, site of the primary carcinoma, metastatic locations within the hand and affected appendage or appendages, the treatment given, and the patient’s length of survival. Men were almost twice as likely to experience acrometastasis as women, and the median age of the patients overall was 58 years (range: 18 months–91 years). Lung, kidney, and breast carcinoma were the three most prevalent primary diagnoses reported in the literature. Cancers of the colon, stomach, liver, prostate, and rectum affected the remainder of the population.

Overall, the right hand was more often host to the metastatic lesions. In addition, almost 10% of the patients experienced lesions in both hands. The third finger was the digit most affected by osseous metastases reported in the literature. Lesions of the thumb, fourth finger, second finger, and fifth finger were less commonly reported. The region of the digit most often affected within the patient population was the distal phalanx. The metacarpal bones, proximal phalanges, and middle phalanges comprised the remainder of the four most frequent acrometastatic sites. In the literature, single lesions were more prevalent than multiple bony lesions.

Based on the reported cases, amputation appeared to be the preferred method of treatment. Radiation, excision, and systemic therapy were the next most frequently used treatments. Patient survival was not well documented within the literature. However, the median survival of patients in the reported cases was 6 months. Thus, our review suggested that a diagnosis of hand metastasis is an indication of poor prognosis.

This report serves to emphasize the importance of properly diagnosing acrometastases. Identifying and effectively treating these metastases in a timely manner can lead to a dramatic improvement in a patient’s quality of life.

## 1. CASE HISTORIES

### 1.1 Case 1

**FIGURE 1:**
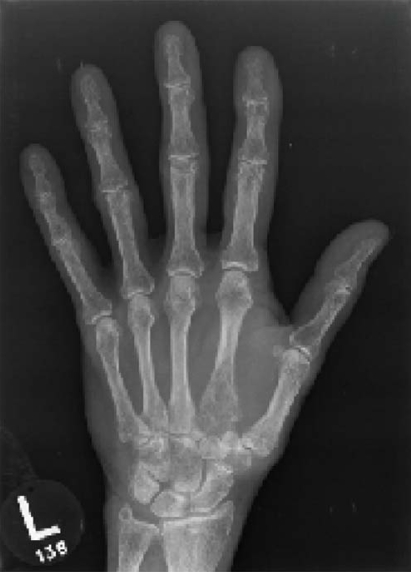
Metastatic bronchogenic carcinoma in the second metacarpal base. Radiograph of the left hand reveals a large, osteolytic, permeative, sightly expansile lesion in the base of the second metacarpal extending from the subchondral bone to the metacarpal shaft, with associated soft-tissue extension.

A 78-year-old woman presented with cachexia, decreased cognition, and left hand pain. The initial suspicion was that her symptoms were the result of an infection. Physical examination revealed a slightly tender elevated area on the back of the patient’s left hand. Radiographs revealed the presence of a permeative osteolytic lesion in the proximal left second metacarpal (Figure 1). This lesion extended from the epiphysis to the midshaft, with cortical perforation and soft-tissue extension. There was no evidence of pathologic fracture. A biopsy of the left hand lesion suggested a diagnosis of non-small-cell lung carcinoma with secondary osseous metastases. A computed tomography scan of the chest revealed a large right hilar mass. In addition, several osteolytic lesions were found in the pelvis, left ilium, left sacrum, right anterior superior iliac spine, and the cortex of the proximal medial femur.

A single 8-Gy fraction of palliative radiotherapy was concomitantly delivered to the patient’s left hand. The treatment proved to be successful: the woman soon experienced pain relief and regained the use of her hand. For example, the patient was not able to hold even a fork with her left hand before radiotherapy. One month after radiotherapy completion, however, she was able to lift three plates with this hand. At the time of writing, 8 months later, the patient was still alive, but she had recently been diagnosed with brain metastases. Her left hand was still pain-free, and she continued to demonstrate an impressive range of motion.

### 1.2 Case 2

**FIGURE 2:**
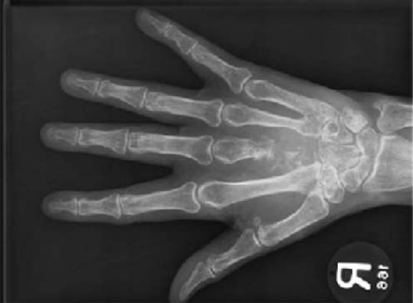
Metastatic breast carcinoma in the third metacarpal and base of the fifth metacarpal. Radiograph of the right hand reveals extensive lytic destruction of the proximal two thirds of the middle metacarpal plus permeative lytic change in the base of the fifth metacarpal.

A 65-year-old woman presented with pain in the distal portion of her right forearm and swelling of the dorsum in her right hand. She had previously ignored these symptoms, because she believed that the swelling stemmed from her chemotherapy injection site. (She had been diagnosed with carcinoma of the breast in 1990.) A radiograph confirmed the presence of extensive lytic destruction involving the proximal two thirds of the third metacarpal (Figure 2). There was also evidence of focal lytic destruction of the distal margin of the capitate and the base of the fifth metacarpal. A lytic lesion was found in the midshaft of the ulna. This woman also had rib metastases.

The patient was treated with a single 8-Gy fraction of radiation to her hand. Seven months after her radiation treatment, the patient remained pain-free and claimed to have regained full use of her right hand.

## 2. DISCUSSION

Acrometastasis to the hands is not common, accounting for approximately 0.1% of all metastatic osseous involvement 1. Handley was the first to report this unusual manifestation in 1906 2. His initial report discussed the case of an elderly woman whose carcinoma of the breast had metastasized to multiple metacarpal bones 2. Because cancer patients are experiencing increased longevity, there is a greater opportunity for metastases to develop throughout the body 3. However, because of the uncommon presentation of secondary disease in the hands, it is difficult for physicians to consider the possibility of acrometastasis when diagnosing unusual hand conditions. In general, the lesion will appear similar to that of an infection, because patients often present with pain, redness or discolouration, tenderness, heat, swelling, erythema, or loss of function 4–6.

The mechanism responsible for the deposition of metastatic tumour cells within the hand is unclear, but an increase in blood flow or a trauma has been suggested in the past 3. Healey and colleagues reported that most patients they encountered with acrometastasis acquired the lesions in their dominant hand 3. That finding was assumed to be a result of the dominant hand being the one that receives a larger quantity of blood and possibly being the one more likely to undergo injury than the non-dominant hand 3. That hypothesis echoed Joll’s concept of trauma-induced acrometastasis, given that he had previously described how repeated trauma may degrade the resistance of surrounding tissue, allowing tumour emboli to settle and grow within the skeletal tissue 7. More recently, it has been suggested that the chemotactic factors (prostaglandins) that are released following a traumatic experience may be responsible for cell migration and adherence to osseous material 5. That theory continues to support the notion that acrometastasis may be a result of a preceding physical injury. It should be noted that both of the women reported here were also affected in the dominant hand.

Bone metastases usually develop in areas rich in red marrow 8. The scarce quantity of this tissue within the bones of the hand supports findings in the literature suggesting that secondary lesions arising in the terminal regions of the extremities are quite remarkable 8,9. Primary lung tumours comprise almost 50% of all cancers that metastasize to the hands 10. That finding is believed to be a result of the use of the systemic arterial system rather than the lymphatic system for distributing these particular tumour cells 10. Other visceral tumours are less likely to result in acrometastases because their primary emboli do not reach the systemic arterial system until after they have passed through the capillary bed of the liver or the lung 11.

Because acrometastases generally accompany widespread disease, the prognosis of patients with acrometastasis is poor 12. Upon presentation of a meta-static hand lesion, patients are anticipated to survive merely 6 months 13. This expectation means that pain palliation is often the primary objective of treatment in these individuals 11. The status of the patient, localization of the lesion, and primary cancer site all dictate the treatment that the oncologist should use 9. Amputation, radiotherapy, curettage, cementation, chemotherapy, and wide excision are the forms of treatment used most often 4,9,12.

### 2.1 Literature Review

An extensive review of the literature generated 257 cases of acrometastasis. Table I summarizes the articles included. Articles were excluded if they were reported in a language other than English and French, unless an English abstract was provided. Articles were analyzed by age and sex of the patient, site of primary carcinoma, metastatic locations within the hand and affected appendage or appendages, treatment given, and the patient’s length of survival.

Based on the articles analyzed, men are almost twice as likely to experience acrometastasis as women. We found 155 cases of men with hand lesions as compared with 84 cases involving women. This finding has previously been acknowledged in the literature, with Asencio and colleagues reporting in 1982 that the sex balance is likely related to the primary cancer site most often responsible for acrometastasis 10. It had previously been reported that lung is the origin of 47% of all cancers that metastasize to the hands 10. Because of the strong relationship between cigarette smoking and the development of lung carcinoma, it is likely that men would experience a greater proportion of acrometastasis 10. Men have been reported to engage more frequently in habitual smoking behaviour and thus would be anticipated to develop lung cancer and eventually secondary hand lesions more frequently than women do 10.

The median age of patients affected by acrometastasis in our literature sample was 58 years, with a range of 18 months to 91 years. Within this population, 113 cases of hand metastases originated from lung cancer, comprising 44% of the cases. Lung was the number one primary cancer site within the sample. A kidney primary was responsible for the second greatest number of acrometastatic cases in the study population. The 31 patients with primary kidney carcinoma contributed 12% of the sample. Lastly, 26 cases of breast cancer resulted in the development of osseous hand lesions. These findings are similar to those previously reported within the literature, although breast carcinoma has been reported to provide equivalent or greater numbers of acrometastatic cases than cancer of the kidney does 5,14,15. Patients with breast as the primary cancer site constituted 10% of the sample. The remaining cases originated from colon, stomach, liver, prostate, rectum, and numerous other cancers.

The right hand was more often involved than the left in the literature sample: 102 cases of osseous lesions were reported to have developed in the right hand as compared with only 84 cases in the left. That finding is consistent with the hypothesis that metastatic hand lesions occur within the dominant hand, because more members of the population are right-handed. Nonetheless, reports acknowledged 23 cases in which both hands were involved.

With 68 cases reported in the publications analyzed, the third finger was the digit most affected by osseous lesions. Next most frequent were the thumb (53 cases), the fourth finger (37 cases), the second finger (35 cases), and the fifth finger (25 cases). The distal phalanx was the region of the digit most affected by bone metastases. This area of the hand was the site of 74 secondary lesions. The metacarpal bones (56 cases), proximal phalanges (26 cases), and middle phalanges (16 cases) comprised the remainder of the four most frequent acrometastatic sites in the sample. Those findings are quite consistent in the literature 8,15,16. Regions of the body with a reduced circulatory speed are preferential for secondary tumour growth 10, which may explain the greater prevalence of lesions within the phalanges of the hand.

Single lesions occurred in 74% of the cases analyzed in the literature review. Again, this finding is consistent with the main primary cancer sites from which the lesions originated. Bronchial carcinomas are typically osteolytic, metastasizing to a single bone within the hand 17. Conversely, breast cancer metastases are sclerotic, lytic, or mixed, and often lead to multiple bony lesions 17. This difference is nicely demonstrated by the two cases presented in this report.

Amputation was the preferred method of treatment in the reported cases. Although treatment was not well reported in the published literature, 50 cases reported complete or partial amputation of the digits. Radiation (30 cases), excision (15 cases), and systemic therapy (10 cases) were the next most frequent treatments. Because amputation is recommended for pain palliation of terminal lesions in the hand, and the distal phalanges are the area most affected, it is quite reasonable that these findings were observed 12. The remaining treatments are consistent with proximal lesions 12. Both women reported in this paper presented with lesions of the metacarpal bones, and so radiation was an appropriate treatment selection. Radiotherapy served to relieve pain and to permit the patients to regain complete use of their hands. Thus radiotherapy is an effective and noninvasive treatment that improves a patient’s quality of life.

Survival was not always reported in the published literature. However, based on the cases that reported this outcome, the mean survival of the patients was 6 months. That finding reinforces the results from Hsu *et al.,* who stated that the average length of survival in patients with hand metastases was merely 6 months 13. Thus, our extensive literature review confirmed that a diagnosis of hand metastasis is an indication of poor prognosis.

## 3. CONCLUSIONS

The present report serves to emphasize the importance of properly diagnosing acrometastases. The presence of cancerous lesions within the hands not only implies severe prognostic implications, but also a very treatable devastation to the patient’s independence. Identifying and effectively treating theses metastases in a timely manner can ensure a dramatic improvement in the patient’s quality of life.

## Figures and Tables

**TABLE I:** Characteristics of cases of acrometastasis to the hands in the literature 1–12,14–167

Characteristic	Value
Total cases	257
Sex [n (%)]	
Male	155 (60)
Female	84 (33)
Unknown	18 (7)
Age (years)	
Median	58
Range	18 months–91 years
Primary cancer site [n (%)]	
Lung	113 (44)
Kidney	31 (12)
Breast	26 (10)
Colon	15 (6)
Other	67 (26)
Unknown	5 (2)
Hand [n (%)]	
Right	102 (40)
Left	84 (33)
Both	23 (9)
Unknown	48 (19)
Digit {*n=*247 [n (%)]}^a^,^b^	
Thumb	53 (21)
Second (index)	35 (14)
Third (middle)	68 (28)
Fourth (ring)	37 (15)
Fifth (little)	25 (10)
Unknown	29 (12)
Metastasis location {*n=*351 [n (%)]}^a^	
Distal phalanx	74 (21)
Metacarpal	56 (16)
Proximal phalanx	26 (7)
Middle phalanx	16 (5)
Unspecified phalanx	17 (5)
Other	53 (15)
Unknown	109 (31)
Type of lesion [n (%)]	
Single	191 (74)
Multiple	61 (24)
Unknown	2 (1)
Treatment {*n=*272 [n (%)]}^a^	
Amputation	50 (18)
Radiation	30 (11)
Excision	15 (6)
Systemic therapy	10 (4)
Other	7 (3)
None	9 (3)
Unknown	151 (56)
Survival (months)	
Mean	6

a Patient may have more than one affected digit, site of metastasis, or treatment option.

b In some patients, acrometastasis affected other areas of the hands.
